# A rare primary hepatic neuroendocrine tumour with laparoscopic resection: a case report

**DOI:** 10.1186/s13256-023-03993-z

**Published:** 2023-06-30

**Authors:** Hanan M. Alghamdi

**Affiliations:** Department of Surgery, College of Medicine, Imam Abdulrahman Bin Faisal University, Al Khobar, Saudi Arabia

**Keywords:** Neuroendocrine tumour, Liver neuroendocrine tumour, Liver primary neuroendocrine tumour

## Abstract

**Introduction:**

Primary hepatic neuroendocrine tumours (PHNETs) are a rare form of hepatic neoplasms, and it is difficult to differentiate them from common hepatic malignancies in routine imaging studies.

**Presentation of the case:**

We describe the case of a 60-year-old Indian male patient with a tentative preoperative diagnosis of hepatocellular carcinoma (HCC). Nevertheless, the definitive post-operative diagnosis was made by Histopathological and immunohistochemical assessment, which revealed a grade II neuroendocrine tumour (NET) of moderate differentiation. Surgical resection was performed through a minimally invasive approach with a favourable postoperative course and a short hospital stay. One-month Post-operative Octreotide scan showed no extrahepatic primary origin of the tumour.

**Discussion:**

PHNET is a rare entity, and multi modalities investigations, including imaging, serology, endoscopy series, and histopathology findings, aside from long-term follow-up to rule out another primary origin, are essential for the final diagnosis of PHNET. Surgical resection stands as the mainstay of treatment of PHNETs.

**Conclusion:**

The absence of primary liver diseases should expand our possible differential diagnosis. Laparoscopic surgical resection of PHNETs carries a favourable outcome.

## Introduction

Neuroendocrine tumours are uncommon and originally derived from neuroendocrine cells distributed widely throughout the body. Theoretically, the tumours can be found in different parts of the body. However, more than 50% of these tumours originate from the gastrointestinal tract or the bronchopulmonary tree in 30% of the cases. Other rare sites include the pancreas (2.5%), reproductive system (1%), biliary tract (1%), and other locations [1, 2].

Edmondson reported the first case of primary NET of the liver in 1958 [3]. The liver is the most common site of NET metastases. It is extremely rare as a primary hepatic NENs, in as few as 0.3% of all NETs and 0.28% to 0.46% of all malignant liver tumours [4, 5].

Around 200 cases were reported in the medical English literature [2–17]. This scarcity of cases, non-specific presentation of the tumour, and mimicking the common liver malignancies in radiological appearance led to misdiagnosis with other hepatic lesions such as HCC, cholangiocarcinoma, or hepatic metastasis [18]. Moreover, PHNETs share common histopathological features with those of other gastroenteropancreatic NETs. Therefore, strict exclusion of hepatic metastases from other NETs is essential for an accurate final diagnosis of PHNETs [3, 12].

Here, we report a rare case of PHNET initially suspected to be HCC before the resection.

## Case presentation

A 60-year-old Indian man with a history of hypertension and hyperuricemia; presented to our hospital initially with a clinical and laboratory picture of obstructive jaundice, for which he underwent ERCP (Endoscopic retrograde cholangiopancreatography), sphincterotomy, and common bile duct (CBD) stent for a distal CBD stricture which was biopsied. The histopathological assessment showed no high-grade dysplasia or malignant cells.

The patient had no other positive findings in his past surgical (including no previous appendectomy), medical, family, psychosocial, or genetic history.

**Fig. 1:**
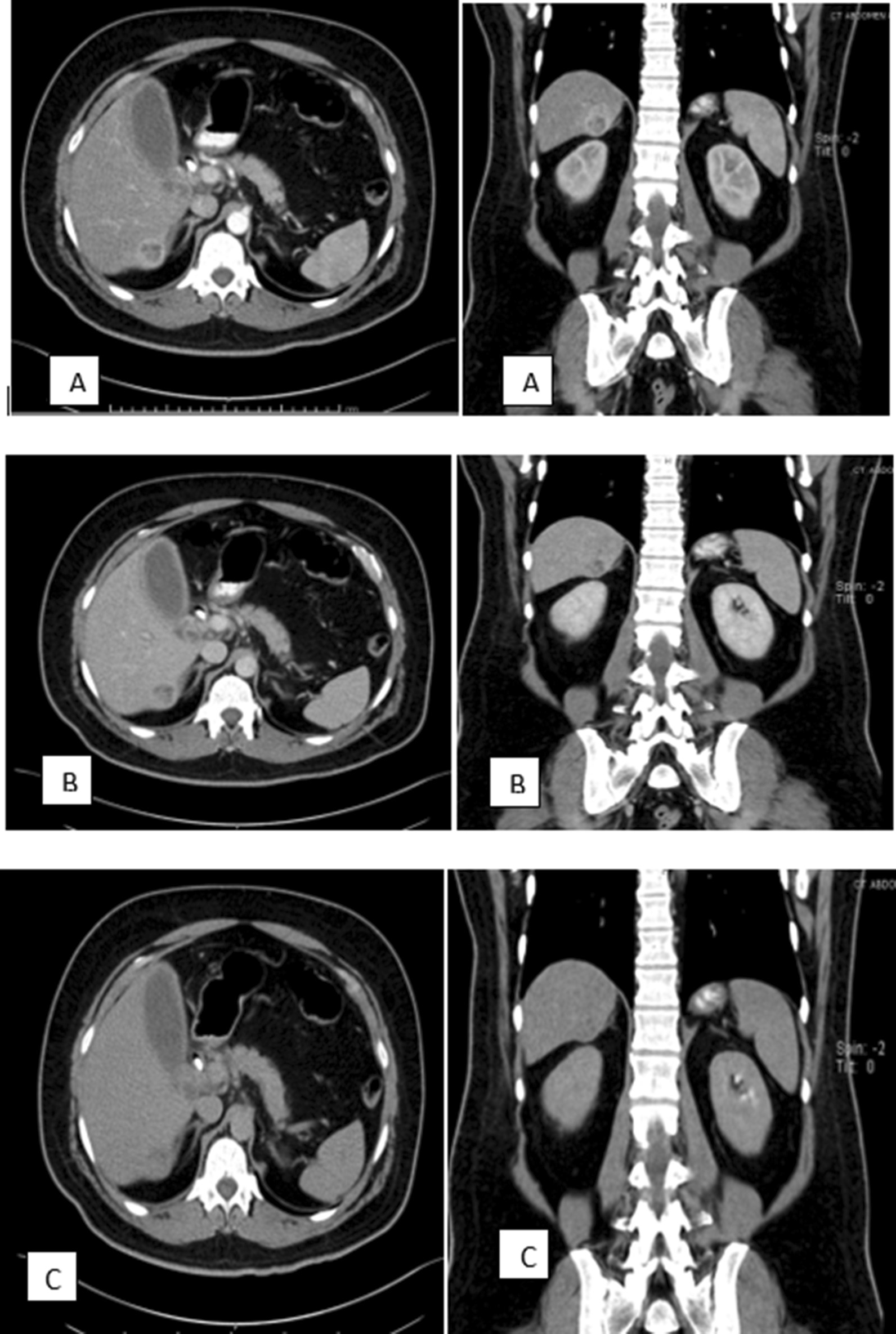
Computerized tomography scan of abdomen and pelvis with contrast; **a** arterial phase, **b** venous phase, **c** delayed phase (axial view on the left side and coronal view on the right side). The mass is seen At segment VI of the RT liver lobe, causing a contour bulge of the liver capsule inferiorly with a clear fat plane between the mass and the RT kidney and is enhancing at the arterial and venous phases (**A**, **B**) with washout at the delayed phase (**C**)

Diagnostic imaging procedures showed incidental right hepatic lobe mass (segment VI) with exophytic growth, which was fairly defined by the CT scan and was located in segment VI with the dimensions of 4 cm (anterior–posterior) by 3 cm (width) by 3.5 cm (height). The mass was iso- to hypodense compared to the liver parenchyma with a central hypoattenuating area representing mainly breakdown. No evidence of calcification or haemorrhage was seen. The mass was causing a contour bulge of the liver capsule inferiorly into the adjacent fat. However, there was a clear fat plane between the mass and the right kidney After IV contrast administration. The mass is seen enhancing at the arterial and venous phases (Fig. 1A, B) with washout at the delayed phase (Fig. 1C). A Well-differentiated HCC was a high clinical suspicion (Fig. 1).

**Fig. 2:**
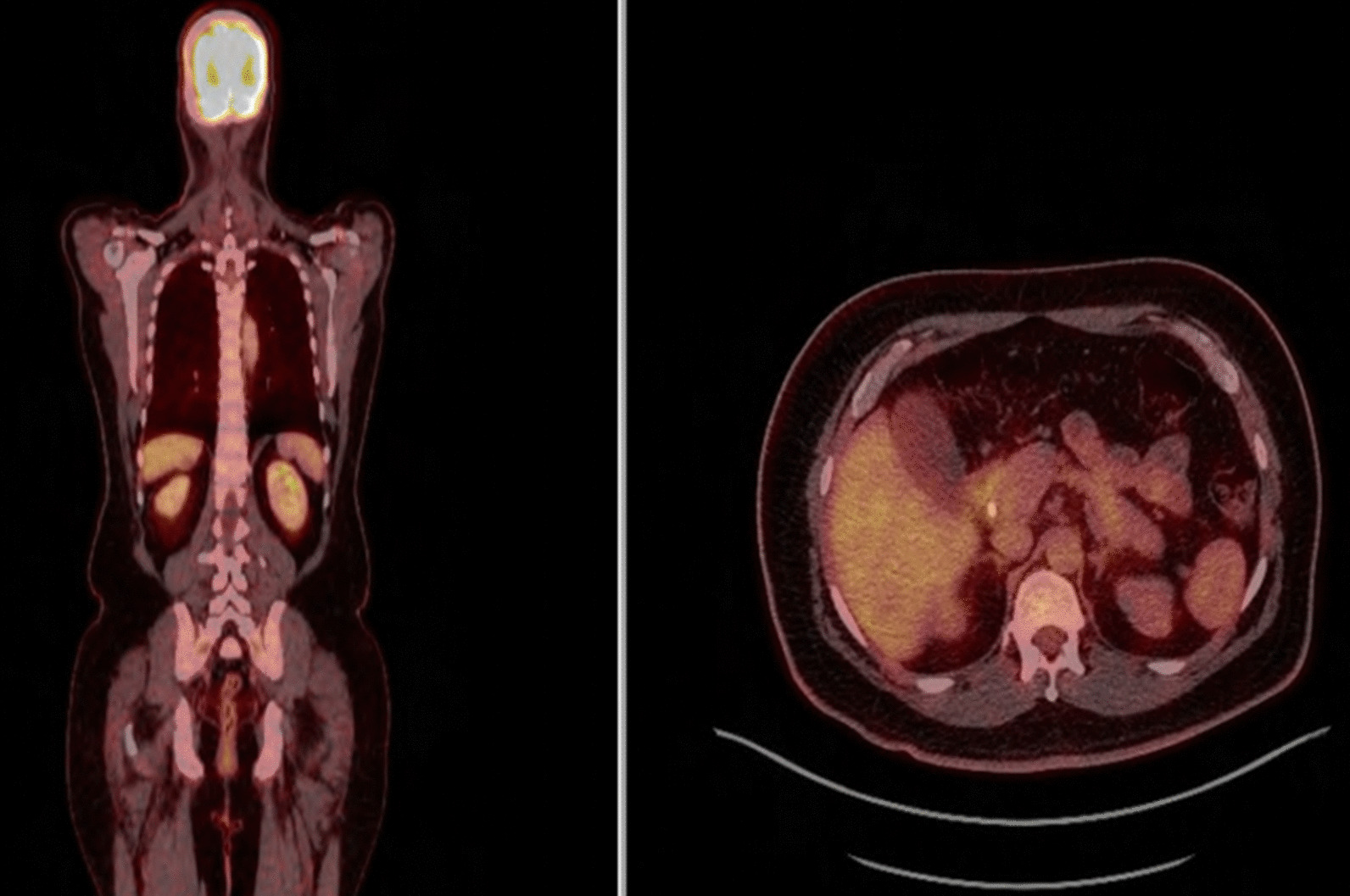
F18 FDG Positron emission tomography-computed tomography images of the coronal liver fusion (left side), Axial fusion (right side). A single hypometabolic hepatic lesion was seen in the right liver lobe (4 × 3 × 3.5 cm^3^). No hypermetabolic lesions were identified in other organs

PET CT scan for Systemic staging showed no evidence of metastasis or another primary site for the tumour (Fig. 2). Moreover, the right hepatic mass showed metabolic activity more diminutive than the adjacent hepatic parenchyma, especially in its centre with SUV_max_ 2.8 (SUV_max_ of adjacent normal hepatic parenchyma = 4.27). In the delayed study, the FDG uptake of the normal hepatic parenchyma has decreased to reach an SUV_max_ of 3.8. The hepatic focal lesion showed a mild increase in activity to reach SUV max of 2.98 metabolic activity, in correlation with the post-contrast CT revealed LI-RADS 5 lesion. The whole picture suggested a probably well-differentiated hepatocellular carcinoma LR-4 per the Liver Imaging Reporting and Data System (LI-RADS® version 2017).

Percutaneous CT-guided true-cut biopsies of the liver lesion were obtained for histopathological assessment, which, unfortunately, was non-confirmatory and showed distorted glands and surrounding fibrosis.

**Fig. 3:**
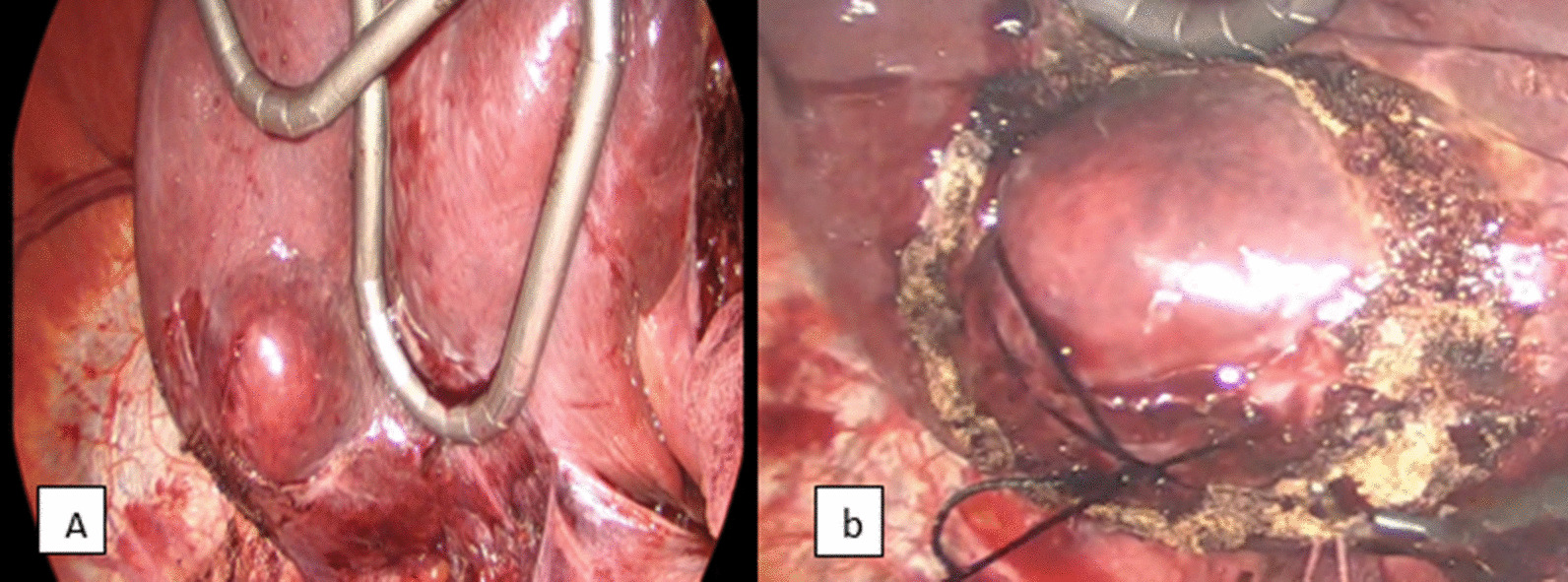
Shows the exophytic component of the tumour at the undersurface of the cephalad retracted liver (**a**), silk suture, a figure of eight fashion was taken deeply to the tumour to aid the counter-traction (**b**)

The patient, therefore, underwent laparoscopic non-anatomical tumour resection with a margin. The tumour was laparoscopically accessible with an exophytic component under the surface of the right liver (Fig. 3). Both monopolar and ultrasonic sealant devices were used for the resection. These electric devices achieved good hemostasis. It was ensured by applying the hemostatic surge-snow product to the operative bed. One tube drain was inserted and removed on the second postoperative day before the patient was discharged.

**Fig. 4:**
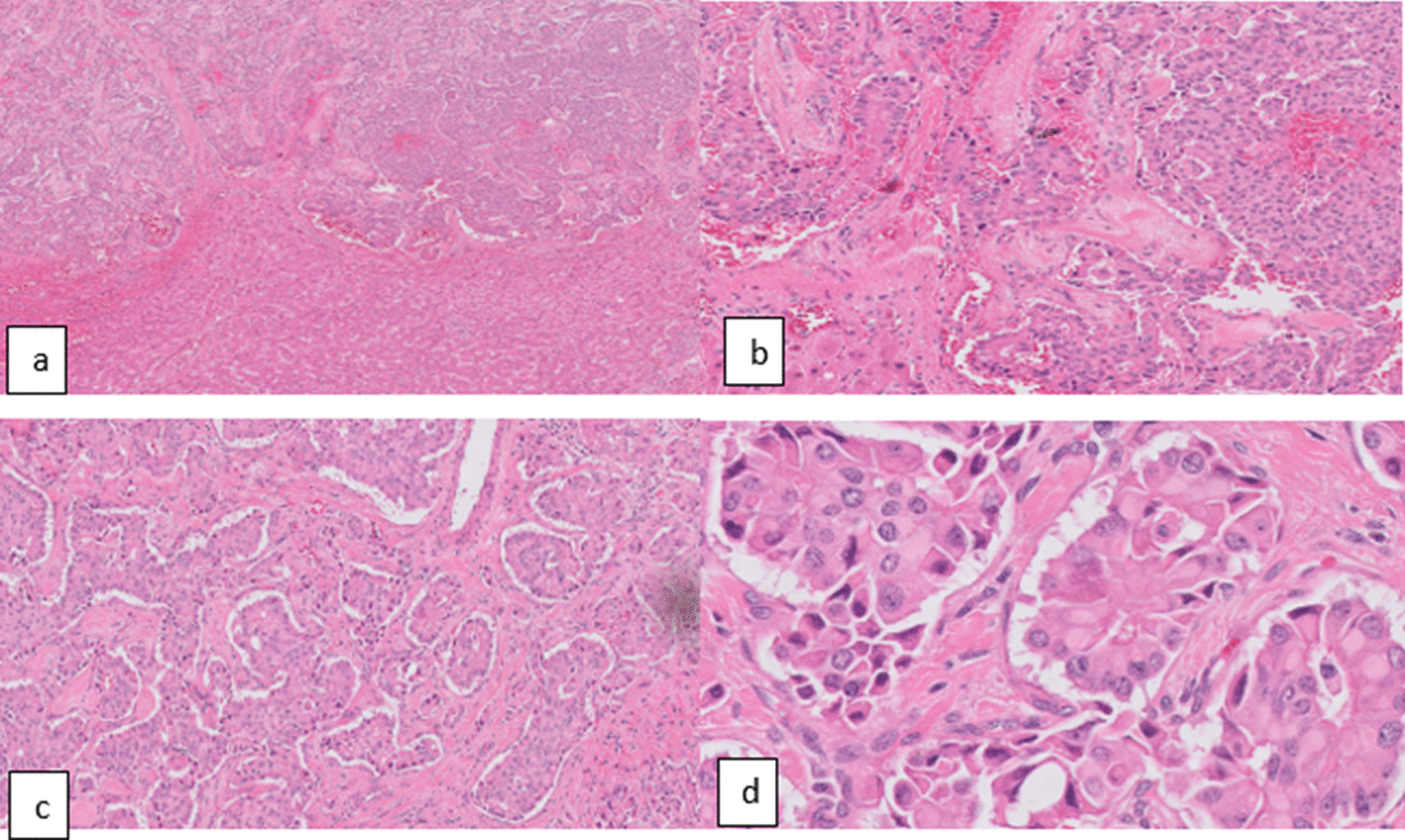
Microscopic picture of the tumour H&E shows tumour cells arranged as solid nests, cells with atypia, varying sizes, multiple mitosis rates, and with large, round, or oval nuclei, fine, granular chromatin, and lying to one side, neuroendocrine tumour-infiltrating the hepatic parenchyma. Magnification: *D* = × 40 (**a**), × 100 (**b**), × 200 (**c**), × 400 (**d**)

Cytopathological examination revealed a single greyish-brown fragment measuring 4.5 × 3 × 3.5 cm^3^ showing a greyish-white cut surface with hemorrhagic contents. It was mixed forming with periductal infiltrate without perineural or lymphovascular invasion (Fig. 4). IHC staining revealed that neoplastic cells are positive for CK (Ventana clone AE1/AE3), synaptophysin (Ventana clone SP11), CD56 (Ventana clone MRQ-42), CK19 (Dako clone RCK108), EMA (Dako clone E29). And negative for Hepatocyte (Dako clone OCH1E5), CK7 (Ventana clone SP52), CK20 (Ventana clone SP33), CEA (Dako clone Monoclonal/II-7), Chromogranin A (Ventana clone LK2H10), GATA-3 (Ventana clone L5-823). The Ki67 proliferation index (Dako clone MIB-1) was 5%. The picture is consistent with a Neuroendocrine tumour, grade 2, moderately differentiated, T1aNxMx.

Upper and lower GI endoscopy series were performed and showed no suspicious pathology. Serum Chromogranin level was checked at 3, 6 months post-surgery and was in the normal range. Octreotide scanning at 6 months revealed no other intra or extrahepatic lesions. However, the patient had no complaints after six months of follow-ups.

## Discussion

NETs arise from cells in the neuroendocrine system and, therefore, were identified in different body systems. Nonetheless, the pathogenesis of PHNETs and their origin is yet not understood [10]. Because therapeutic strategies are significantly determined by understanding how PHNETs occur, several hypotheses explain their origin. Those include the suggestions that they arise from malignant liver stem cells that are differentiated into neuroendocrine cells, ectopic adrenal or pancreatic tissues within the liver, or intrahepatic biliary duct epithelium [10, 15]. The theoretical hypothesis of biliary duct epithelial origin seems to be more acceptable because the biliary epithelium contains argentaffin (neuroendocrine) cells, and chronic inflammation of the biliary system could cause metaplasia to intestinal cells, which may, in turn, proliferate to NET [15, 19].

The clinical presentation of PHNETs differs from other NETs. They are often discovered incidentally because they are commonly present as an endocrinologically nonactive hepatic mass. In our presented case, the PHNET was asymptomatic. Although the initial presentations were symptoms of obstructive jaundice, the size and location of the tumour could not cause the pressure effect that led to this presentation. Moreover, the concomitant benign stricture of distal CBD clearly explained the manifestations of obstructive jaundice resolved after CBD stenting.

Only 5% of hepatic NETs present as classical carcinoid syndrome with flushing, diarrhoea, and abdominal pain and are mostly in metastatic liver NETS [3, 20].

While PHNET does not exhibit obvious carcinoid syndrome-related symptoms, the patient presents with non-specific and vague clinical presentation symptoms such as vague abdominal pain, distension, epigastric discomfort, loss of appetite, fatigue, jaundice, weight loss, and a palpable mass in the right upper quadrant which are often present as the tumour enlarges. On many occasions, the tumour is discovered incidentally during investigations for other reasons. Owing to this, early detection of PHNETs is often difficult [11, 16].

Despite radiographic imaging advances, diagnosis cannot be made preoperatively by the current technology [15, 19]. Moreover, since these lesions exhibit no specific features in CT or MR imaging studies, unlike HCC, which has a typical radiological pattern of marked arterial enhancement with a pathognomonic portal and delayed phases washout, which cannot be confounded like the lack of specific radiological feature for PHNET [5, 8, 11, 13].

The preoperative CT scan misidentified the tumour as HCC in this reported case because of similar radiological features. Similarly occurred with PET/CT scan study.

The role of PET-CT scans is to exclude the presence of extrahepatic lesions [4, 13]. However, it demonstrates a low density on the lesion site and a contrast enhancement in the arterial phase, which is inconclusive for diagnosis.

Due to the presence of somatostatin receptors in NET, the Octreotide scintigraphy is very effective in confirming the Primary and distant metastasis carcinoid tumours compared to CT scan and or MRI) with a sensitivity of 90% and specificity up to 83% [18]. Prognostic test in the follow-up mainly by Measurement of chromogranin A (CgA) plasma level and Octreotide scanning. This highly diagnostic modality cannot be employed in PHNETs since post-resection histopathology studies discover the diagnosis due to the silent presentation of this tumour from the classic carcinoid syndrome.

Common Tumor markers for gastrointestinal and hepatic tumours, like Alpha-Fetoprotein (AFP), Carcinoembryonic Antigen (CEA), and CA19-9, are commonly negative [10, 11, 15]. Only serum Chromogranin A can be used as a marker in diagnosing and following NET [15, 18, 25].

In our case, Tumor markers (AFP, CEA, and CA19-9) were in the normal range, post-operative serum CgA was normal, and the octreotide scanning six months after the surgery showed no evidence of another primary tumour. In our case, the preoperative needle biopsy was inconclusive, and the diagnosis was achieved only after surgical resection.

While the definite diagnosis of hepatic NET depends mainly on pathology and immunohistochemistry, The accuracy of preoperative needle biopsy is still questionable because of confusing hepatic NETs with other liver tumours, particularly HCC and CCC [11, 15]. For instance, in Prosser's histopathologic study [21], the preoperative biopsy of 23 confirmed hepatic NETs was misdiagnosed in nine cases (39%) as HCC or adenocarcinomas**.** Therefore, a preoperative needle biopsy is not definite. Histopathological and immunohistochemistry cannot distinguish between the PHNET and metastatic NET to the liver. Thus, other modalities by applying CT, MRI, Octreotide scan, PET, gastrointestinal endoscopy series, and long-term follow-up are necessary to make the final diagnosis of PHNET.

Pathological features of hepatic NETs generally appear in the macroscopic picture as a grey-yellow in colour and well-demarcated lesion with multiple irregular hemorrhagic areas but with cystic components occasionally [15, 23]. Microscopically, the tumour exhibits unique findings of an insular, nested, trabecular, or mixed pattern of cell growth pattern [14, 15].

The immunobiological study of the tumour markers typically demonstrates positivity of neuron-specific enolase (74.1–90%), CgA (66.7–95%), and synaptophysin (48.9–91.7%) and CD56 and cytokeratin [14, 15, 23].

The most recent WHO classification and grading for neuroendocrine neoplasms (NENs) of the gastrointestinal tract and hepatopancreatobiliary organs, published in August 2019 [22], categorises neuroendocrine neoplasms according to their differentiation into well-differentiated tumours and poorly differentiated carcinomas. Then the poorly differentiated carcinoma is subcategorised into low grades (1), intermediate grade (2) and high grades (3), depending on mitotic rate and ki-67 index. For tumours composed of mixed neuroendocrine and non-neuroendocrine elements, a new diagnostic term MiNEN: Mixed neuroendocrine–non-neuroendocrine neoplasm introduced [22, 23, 25]. The tumour of our presented case fits the criteria of G2 NETs with moderate differentiation.

PHNET is usually a solitary lesion and is best treated with surgical resection as the first line of treatment, with 5-year survival rates of more than 75%. In cases with multiple liver lesions or a huge tumour size that may leave no adequate liver tissue after resection, the liver transplant might be considered the only surgical cure [14, 15].

Non-surgical treatment, including RFA (radiofrequency ablation), TACE (transarterial chemoembolization), chemotherapy, and combination therapy, can be chosen for selected patients with metastasis and unrespectable tumours. These treatment modalities relieve the clinical symptoms and inhibit the growth of tumours. However, their effectiveness is inferior to surgical treatment [6, 17, 24].

## Conclusion

Diagnosis of PHNETs is quite challenging, and its preoperative misdiagnosis is reported. The diagnosis is only confirmed by histopathological and immunohistochemical assessment along with a wide variety of diagnostic workups to rule out extrahepatic primary tumour sources. The mainstay of treatment is surgical resection which can be performed through a minimally invasive approach in selected cases.

The preoperative misidentification of liver HCC requires considering PHNETs in the differential diagnosis of solitary liver solid lesions.

## Data Availability

Available.

## Abbreviations

PHNETs: Primary hepatic neuroendocrine tumour
HCC: Hepatocellular carcinoma
NET: Neuroendocrine tumour
ERCP: Endoscopic retrograde cholangiopancreatography
CBD: Common bile duct
AFP: Alpha-fetoprotein
CEA: Carcinoembryonic antigen
RFA: Radiofrequency ablation
TACE: Trans arterial chemoembolization
MiNEN: Mixed neuroendocrine-non-neuroendocrine neoplasm
Plasma CgA: Plasma chromogranin A

## Declarations

### Research registration

No new device is used, or a new procedure is performed; therefore, it does not apply to this case report.

### Guarantor

By the author who accepts full responsibility for the information and the conduct of the study; I had access to all data and complete control of publishing it.
